# Quantitative proteomics in lung cancer

**DOI:** 10.1186/s12929-017-0343-y

**Published:** 2017-06-14

**Authors:** Chantal Hoi Yin Cheung, Hsueh-Fen Juan

**Affiliations:** 1 Institute of Molecular and Cellular Biology, National Taiwan University, Taipei, Taiwan; 2 Department of Life Science, National Taiwan University, Taipei, Taiwan; 3 Graduate Institute of Biomedical Electronics and Bioinformatics, National Taiwan University, No. 1, Sec. 4, Roosevelt Road, Taipei, 10617 Taiwan

**Keywords:** Quantitative proteomics, Lung cancer, Biomarkers, Drug targets, Functional network

## Abstract

Lung cancer is the most common cause of cancer-related death worldwide, less than 7% of patients survive 10 years following diagnosis across all stages of lung cancer. Late stage of diagnosis and lack of effective and personalized medicine reflect the need for a better understanding of the mechanisms that underlie lung cancer progression. Quantitative proteomics provides the relative different protein abundance in normal and cancer patients which offers the information for molecular interactions, signaling pathways, and biomarker identification. Here we introduce both theoretical and practical applications in the use of quantitative proteomics approaches, with principles of current technologies and methodologies including gel-based, label free, stable isotope labeling as well as targeted proteomics. Predictive markers of drug resistance, candidate biomarkers for diagnosis, and prognostic markers in lung cancer have also been discovered and analyzed by quantitative proteomic analysis. Moreover, construction of protein networks enables to provide an opportunity to interpret disease pathway and improve our understanding in cancer therapeutic strategies, allowing the discovery of molecular markers and new therapeutic targets for lung cancer.

## Background

Lung cancer is the most common cancer-related mortality worldwide, with approximately 27% of all cancer deaths per year [1]. Lung cancer divided into two main types including small cell lung cancer (SCLC) and non-small cell lung cancer (NSCLC). 10–15% of lung cancer cases are SCLC which is responsive to chemotherapy and radiation treatment [2]. However, more than eighty percent of lung cancer is NSCLC, which has become resistant to anticancer drugs [3]. Regardless of subtypes, the overall survival rate of lung cancer patients is still disappointing; less than 7% of patients survive 10 years following diagnosis across all stages of lung cancer [4]. Current treatments and therapies are not sufficient to reduce the mortality for this malignancy. To address this challenge, early detection and systemic therapy might be the solution to alter the mortality trend and gain our knowledge in lung cancer progression. Recent omics researches in lung cancer have been focused on classification of lung cancer, correlation of gene and protein expression, and identification of novel molecular targets [5].

Proteins are involved in all biological processes which can be regarded as the final stage of biological information from genome. Proteomics is extremely dynamic and complex due to the continuous response to the change of environment, drug treatment, and post-translational modification [6]. Large-scale and systematic analysis of proteins is a complete and unique profile for characterization and biological activity. Quantitative proteomics provides the relative different protein abundance in normal and disease samples which offers ultimate information for molecular interactions, signaling pathways, and biomarker identification in human disease research [7]. In addition, the integration of biomarker discovery from different pulmonary diseases and multiple sample types may serve as a valuable resource for future clinical validation studies [8, 9]. To interpret the data generated from high-throughput technologies, a combination of computational and experimental approach is required for analyzing complex interaction of many levels of biological information which may benefit our understanding in biochemical pathways, regulatory networks, and disease therapies in lung cancer [10, 11].

## Development and techniques of quantitative proteomics

**Fig. 1:**
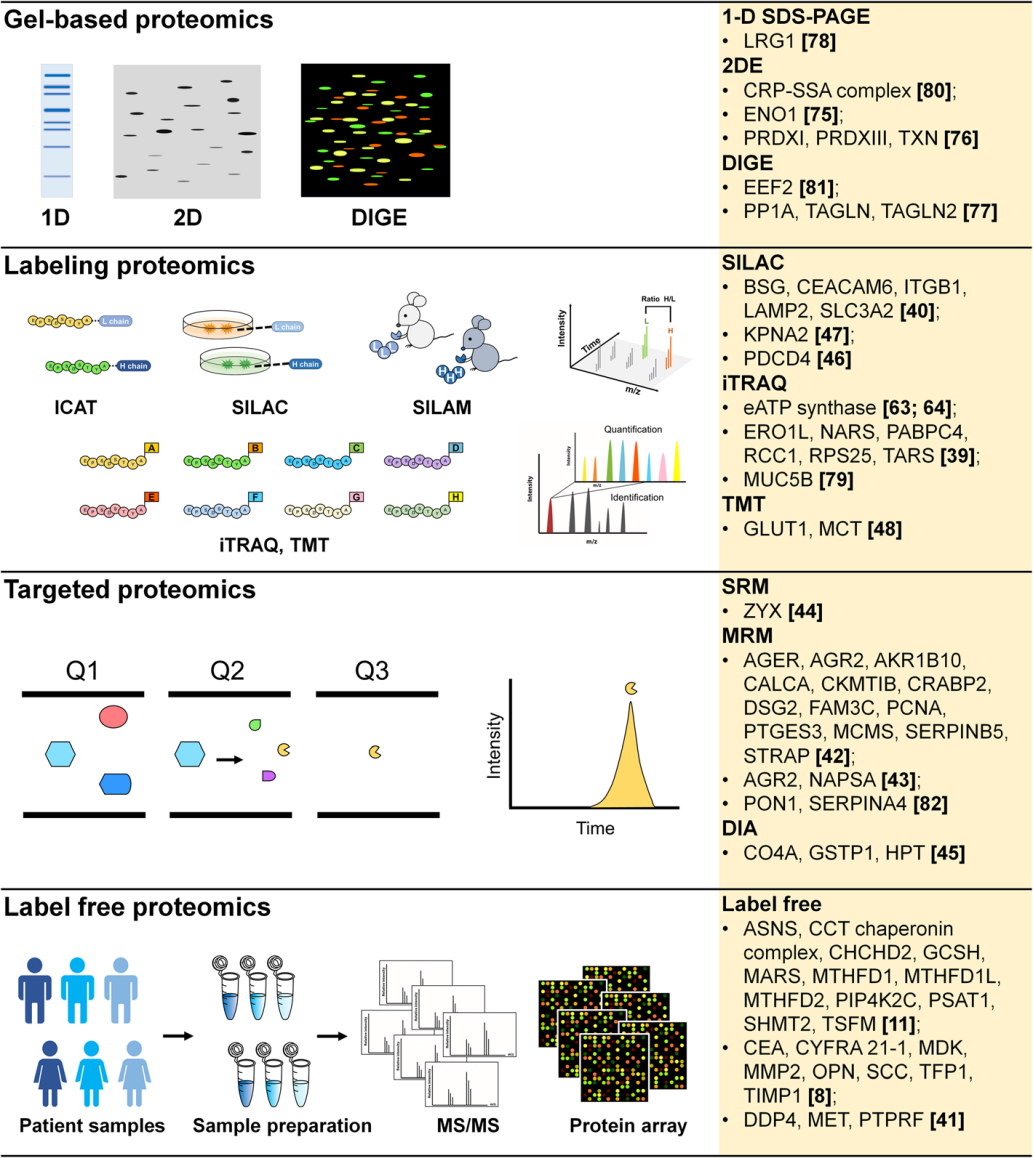
The applications of quantitative proteomics for discovery of biomarkers in lung cancer study. Quantitative proteomics not only provides a list of identified proteins, it also quantifies the changes between normal and disease sample profiles which enables to generate classification models or biomarkers. Biomarkers are measurable biological indicators found in tissue, cells, blood or other body fluids that may be used for detection, diagnosis treatment and monitoring in cancer research by the means of advanced quantitative proteomic approaches: gel-based, stable isotope labeling, targeted proteomics, and label free. In gel-based proteomics, one-dimensional (1D) gel electrophoresis, two-dimensional (2D) polyacrylamide gel electrophoresis, and difference gel electrophoresis (DIGE) approaches have been developed and utilized to separate protein from protein mixtures and identification. In vitro labeling, the peptides are modified by stable isotope labeling (ICAT, iTRAQ, TMT) prior to MS analysis. In vivo labeling, isotope labeling (SILAC and SILAM), specific supplements containing distinct forms of amino acid are given to living cells or mammals prior to MS analysis. The resulting spectrum is able to generate peptide intensity for both identification and quantitation. Targeted proteomics (SRM, MRM, and DIA) using triple quadrupole mass spectrometers systems where the mass of the intact targeted analyte is selected in the first quadrupole (Q1), and then the fragmentation of the Q1 mass-selected precursor ion by collision-induced dissociation in the second quadrupole (Q2), finally a desired product ion is selected in the third quadrupole (Q3), which is then transmitted to the detector. This method of absolute quantitation in targeted proteomics analyses is suitable for identification and quantitation of target peptides within complex mixtures independent on peptide-specific manner. Label-free quantification is an alternative method for samples that cannot directly label and enables the comparison of protein expression across different samples or treatment regardless the number of samples. Protein microarray is another label-free method which is a high-density and high-throughput microarray containing thousands of unique proteins to identify the interactions on a large scale

Proteomics is an analysis of dynamic systems in biology which consists a range of diversity that are insufficient to analyze with any single method. Quantitative proteomics not only provides a list of identified proteins, it also quantifies the changes between normal and disease sample profiles in order to generate classification models. Here, we review quantitative proteomics into four major approaches: gel-based, stable isotope labeling, label free, and targeted proteomics for lung cancer studies (Fig. 1).

## Gel-based and gel-free proteomics

The first and most important step in quantitative proteomic analysis is the separation of a complex protein mixture [12]. Gel-based proteomics including one-dimensional (1D) gel electrophoresis, two-dimensional (2D) polyacrylamide gel electrophoresis, and difference gel electrophoresis (DIGE) approaches have been developed and utilized to separate protein from protein mixtures and identification [13]. 1D- and 2D-PAGE are simple and straight forward in a principle of molecular weight (*M*_r_) and isoelectric point (p*I*)-based separation. DIGE has been developed as a multicolor detection for comparing protein abundance of samples within a single gel, where each protein sample is prelabeled with spectrally distinct fluorescent dye [14]. To reduce inter-gel variation, internal standard is applied for normalization across different gels. However, membrane proteins, low abundance proteins, alkaline proteins, and high molecular weight proteins remain an area of considerable concern in gel-based proteomics [15]. Gel-free proteomic techniques have been developed to fulfill the shortage of gel-base proteomics [13]. Both gel-based and gel-free proteomics are well-established quantitative proteomics which compare the proteome of normal and disease samples in a global aspect which led to magnify the identification of novel protein candidates associated with lung cancer [16].

## Stable isotope labeling proteomics

For quantitative analysis, stable isotope labeling coupled with mass spectrometry (MS)-based techniques is often performed in cancer research [7]. Mass spectrometer is composed of two major compartments: (1) an ionization source that generates ions of target molecules and (2) a mass analyzer that sorts molecules by mass-to-charge ratio (m/z). Stable isotopic labeling methods have been developed and applied in quantitative proteomics as a routine means to analyze protein expression patterns for multiple samples as comparison (Table 1). In vitro labeling, the peptides are modified by stable isotope labeling (ICAT, iTRAQ, TMT, diLeu, and DiART) prior to MS analysis [17–21]. The resulting spectrum is able to generate peptide intensity for both identification and quantitation. In vivo approaches are based on incorporation of isotope label such as SILAC into proteins presenting in living cells that specific media containing distinct forms of amino acid are given [22]. Stable isotope labeling in mammals (SILAM) has been developed by combining ^15^N spirulina with a protein-free chow to overcome the limitation of SILAC to cell culture [23].

**Table 1:** Common isotopic labeling methods in quantitative proteomics

Types of Label	Principles	Comparison	Methods	Year	Ref.
Isotope-Coded Affinity Tags (ICAT)	Thiol group	Pairwise: duplexed	In-vitro	1999	[17]
Stable isotopic labeling with amino acids in cell culture (SILAC)	Metabolic incorporation of lysine or arginine	Pairwise: non-labeled (light); Lys4 and Arg6 (middle); Lys8 and Arg10 (heavy)	In-vivo	2002	[22]
Tandem Mass Tag (TMT)	Free amino group	Multiplex: 2-plex, 6-plex, and 10-plex	In-vitro	2003	[19]
Isobaric Tags for Relative and Absolute Quantification (iTRAQ)	N-terminus and lysine side chains of peptide	Multiplex: 4-plex and 8-plex	In-vitro	2004	[18]
Stable isotope labeling in mammals (SILAM)	combining ^15^N spirulina with a protein-free chow	Pairwise: non-labeled (light); and nitrogen ^15^N (heavy)	In-vivo	2004	[23]
Deuterium isobaric Amine Reactive Tag (DiART)	*N*-terminus and lysine side chains of peptide	Multiplex: 6-plex	In-vitro	2010	[21]
*N,N*-Dimethyl leucine (DiLeu)	*N*-terminus and ε-amino group of the lysine side chain	Multiplex: 4-plex	In-vitro	2010	[20]
Terminal amine isotopic labeling of substrates (TAILS)	neo-N-terminal peptides	Multiplex: iTRAQ-based labeling	In-vitro	2011	[27]
iTRAQ hydrazide (iTRAQH)	carbonylated peptide	Multiplex: 4-plex	In-vitro	2012	[24]
Stable isotope labeled carbonyl-reactive tandem mass tags (Glyco-TMT)	N-linked glycans	heavy/light-TMT labeled glycans	In-vitro	2012	[26]
Irreversible isobaric iodoacetyl Cys-reactive tandem mass tag (iodoTMT)	Cys-redox modifications	Multiplex: iTRAQ-based labeling	In-vitro	2014	[25]

To characterize the post-transcriptional modifications in proteomics, several isobaric reagents were developed for selective labeling such as carbonylated residues and cysteine residues which might expand our knowledge of dynamic system in cancer progression [24–26]. Moreover, a novel iTRAQ-based labeling has been used in distinguishing protease-generated neo-N termini from N-termini of mature protein. This approach can be applied in characterization of post-translational modification [27].

## Label free proteomics

Label-free quantification is an alternative method for samples that cannot directly label, and enables the comparison of protein expression across different samples or treatment regardless the number of samples [28]. Label-free quantification can be divided into two categories: peptide peak intensity based quantification and spectral counting quantification that depends on the number of peptides identified from a protein of interest [29]. Label-free proteome quantification encounters many limitation, several published algorithms are available for additional calculations to compute the predicted abundance of proteins in the sample. Protein microarray is another label-free method which is a high-density and high-throughput microarray containing thousands of unique proteins to identify the interactions on a large scale [30]. Protein microarray has the same concept as DNA microarray that is rapid and automated, moreover, protein microarray also solved the limitation of gene expression levels for proteomics [31]. The probe molecules labeled with fluorophores, chromogen and radioiostopes aim to compare protein expression in different samples [32].

## Targeted proteomics

Selected reaction monitoring (SRM), multiple reaction monitoring (MRM), and data-independent acquisition (DIA) are widely used MS-based proteomics which have been considered as true quantification techniques for targeted quantification of protein [16, 33, 34]. Targeted quantitation using triple quadrupole mass spectrometers systems where the mass of the intact targeted analyte is selected in the first quadrupole (Q1), and then the fragmentation of the Q1 mass-selected precursor ion by collision-induced dissociation in the second quadrupole (Q2), finally a desired product ion is selected in the third quadrupole (Q3), which is then transmitted to the detector [35]. This method of absolute quantitation in targeted proteomics analyses is suitable for identification and quantitation of target peptides within complex mixtures independent on peptide-specific manner [36]. DIA requires no prior knowledge of target peptides and obtains much larger numbers of peptide than SRM or MRM. DIA analysis is a method that all peptides within a given window are subjected to fragmentation, then it is repeated until the mass spectrometer marches up the full m/z range. This powerful targeted proteomics provides accurate peptide quantification without being limited to predefined peptides of interest.

## Applications of quantitative proteomics in lung cancer

Quantitative proteomics allows the discovery of molecular markers and new therapeutic targets for lung cancer. Predictive markers of drug resistance, lung cancer diagnosis, and prognostic markers in lung cancer have also been discovered by quantitative proteomics analysis [37].

## Biomarkers in lung cancer

Biomarker is a measurable biological indicator found in tissue, cells, blood or other body fluids that may be used for detection, diagnosis treatment and monitoring in cancer research [38]. The characterization of specific protein patterns associated with lung cancer as a discovery strategy for biomarker identification in clinical research. Quantitative proteomics reveals several biomarker candidates in lung cancer through comparing differentially expression proteins of lung cancer and normal individual [8]. Biomarkers identified by quantitative proteomics provide valuable information for the researchers to develop better personalized medicine and early and precise diagnostic markers for the lung cancer patients (Table 2).

**Table 2:** Quantitative proteomic studies in lung cancer

Biomarker	Types of Sample	Results	Year	Ref.
ENO1	Lung cancer tissue	ENO1 was consistently up-regulated in all 14 cases of lung cancer, and suggested that basaloid carcinoma is a unique subtype of NSCLC	2004	[75]
PRDXI, PRDXIII, TXN	Lung cancer tissue	Enhanced lung cancer cell survival and proliferation	2006	[76]
PPIA, TAGLN, TAGLN2	Lung cancer tissue	Early diagnostic markers for lung cancer	2009	[77]
AGR2, NAPSA	Lung cancer tissue	Stage-related protein candidates for stage IA and IIIA lung adenocarcinoma	2010	[43]
LRG1	Urinary exosome and lung tissue of NSCLC patient	Non-invasive diagnosis of NSCLC in urine	2011	[78]
AGER, AGR2, AKR1B10, CALCA, CKMTIB, CRABP2, DSG2, FAM3C, PCNA, PTGES3, MCMS, SERPINB5, STRAP	Lung cancer tissue	84–88% of the protein expression differences of SCC and 44 ADC proteins measured by shotgun analyses of the SCC, ADC and normal pools were confirmed in an independent set of specimens	2012	[42]
Ectopic ATP synthase	Lung tumor xenograft and lung cancer cell	Citreoviridin revealed antitumorigenic effects in lung cancer	2012 2013	[63, 64]
MUC5B	Adenocarcinoma tissue	Aberrant expression of MUC5B was identified in 71% of lung adenocarcinomas in the tumor tissue microarray	2013	[79]
ASNS, CCT chaperonin complex, CHCHD2, GCSH, MARS, MTHFD1, MTHFD1L, MTHFD2, PIP4K2C, PSAT1, SHMT2, TSFM	Lung cancer tissue and xenograft	Integrating the omic data from DNA, RNA, and proteins data sets to reveal new anticancer therapeutic targets for lung cancer	2014	[11]
CEA, CYFRA 21–1, MDK, MMP2, OPN, SCC, TFP1, TIMP1	Lung cancer tissue, cell-line, and conditioned medium	Biomarker model was developed which accurately distinguished subjects with lung cancer from high risk smokers	2015	[8]
CRP-SAA complex	Serum	Higher expression of CRP-SAA level was associated with severe clinical features of lung cancer	2015	[80]
DPP4, MET, PTPRF	Pleural effusion (PE)	Diagnostic biomarkers of NSCLC from PE proteome	2015	[41]
GLUT1, MCT	Lung cancer cell line	Quantitative proteomics of TMT labeled SCC and ADC suggested that MCT1 and GLUT1 are the promising drug targets or histological markers	2015	[48]
KPNA2	lung adenocarcinoma cell line	KPNA2-mediated modulation of cell migration in lung cancer	2015	[47]
PDCD4	NSCLC cell	Longer overall survival of lung cancer patients with PTX treatment (personalized medicine)	2015	[46]
ZYX	Plasma	Early diagnostic marker for NSCLC	2015	[44]
BSG, CEACAM6, ITGB1, LAMP2, SLC3A2	Lung cancer-derived exosome	NSCLC-related proteins identified from the study of exosomal proteome as promising candidates	2016	[40]
CO4A, GSTP1, HPT	Bronchoalveolar lavage fluid	More sensitive biomarkers were identified by a DIA-based quantitative proteomic approach from bronchoalveolar lavage fluid	2016	[45]
EEF2	Lung cancer tissue	Clinical tissue studies showed that EF2 protein was significantly overexpressed in LSCC tissues, compared with the adjacent normal lung tissues	2016	[81]
ERO1L, NARS, PABPC4, RCC1, RPS25, TARS	Lung cancer tissue	ERO1L and NARS were positively associated with lymph node metastasis, in which ERO1L overexpression in patient with early stage of adenocarcinoma was associated with poor overall survival	2016	[39]
PON1, SERPINA4	Serum	Meta-markers might have better specificity and sensitivity than a single biomarker and thus improved the differential diagnosis of lung cancer and lung disease patients	2016	[82]

The survival rate of lung cancer patients is highly correlated to the stage of lung cancer; therefore, improve the diagnostic strategies for early lung cancer detection may increase patient survival. Hsu et al. identified 133 protein candidates from paired adenocarcinoma (ADC) tissues with different extents of lymph node involvement by iTRAQ-labeling technology coupled with 2D-LC-MS/MS [39]. They further validated six potential biomarkers (ERO1L, NARS, PABPC4, RCC1, RPS25, and TARS) which were highly expressed in ADC tissues compared to the adjacent normal tissues. In addition, they found ERO1L and NARS are positively associated with lymph node metastasis, in which ERO1L overexpression in patients with early stage of ADC was associated with poor overall survival. Another recent study of triple SILAC quantitative proteomics identified several biomarkers by comparing the protein abundance of immortalized normal epithelial cell derived exosomes and NSCLC exosomes [40]. Integrin beta-1 (ITGB1), Basigin (BSG), 4 F2 cell-surface antigen heavy chain (SLC3A2), lysosome-associated membrane glycoprotein 2 (LAMP2), and carcinoembryonic antigen-related cell adhesion molecule 6 (CEACAM6) are the NSCLC-related proteins identified from their study of exosomal proteome as promising candidates.

Label-free quantitative proteomic analysis was performed and a significant higher protein levels of hepatocyte growth factor (MET), dipeptidyl peptidase IV (DDP4) and Receptor-type tyrosine-protein phosphatase F (PTPRF) in malignant pleural effusion (PE) samples were found comparing to benign and paramalignant PE samples [41]. Proteomic profiling of body fluids presents a sensitive diagnostic tool for early cancer diagnosis and establishes a new database of differential lung tumor-proximal body fluid (PE) proteomes to facilitate the identification of biomarkers for discriminating NSCLC from nonmalignant pulmonary diseases. In a study of shotgun proteomics, Kikuchi et al. identified 3621 proteins from the analysis of pooled human samples of 20 squamous cell carcinoma (SCC), 20 adenocarcinoma (ADC), and 22 control specimens. To further assess the concordance between shotgun proteomics and targeted proteomics on the differentially expressed proteins, they analyzed 20 SCC, 20 ADC and 21 normal tissues by MRM analysis. 84–88% of the protein expression differences (42 SCC and 44 ADC proteins) measured by shotgun analyses of the SCC, ADC and normal pools were confirmed in an independent set of specimens [42]. Moreover, Kawamura et al. identified 81 proteins were associated with stage IA and stage IIIA lung adenocarcinoma by shotgun proteomics using formalin-fixed paraffin-embedded materials, then MRM targeted proteomic quantification was applied to verify for those protein candidates and found that Napsin-A (NAPSA) and anterior gradient protein 2 homolog (AGR2) might be the stage-related protein candidates for stage IA and IIIA lung adenocarcinoma [43].

A recent study applied highly multiplexed liquid chromatography-selected reaction monitoring (LC-SRM) assay to verify the biomarker candidates in plasma samples for lung cancer. A total of 17 proteins were verified as potent tumor markers, especially, a novel plasma-based biomarker, zyxin (ZYX) was identified as a potential early diagnostic marker for NSCLC [44]. Overall, targeted proteomics is able to yield high probability biomarkers for clinical validation in large patient cohorts and represents a strategy to identify and verify novel different types of diseases [36]. Moreover, integrated biomarker discovery from multiple sample types including lung cancer tissues, cell lines and conditioned medium has established to construct a biomarker model (TFPI, MDK, OPN, MMP2, TIMP1, CEA, CYFRA 21–1, SCC) which enables to classify lung cancer patients from high risk smokers [8]. A recent clinical research of the bronchoalveolar lavage fluid (BALF) proteomic analysis by combining a simple pre-treatment and a sequential windowed acquisition of all theoretical fragment ion mass spectra (SWATH) DIA MS approach provided useful resources for the discovery of potential biomarkers for lung disease [45]. BALF is usually discarded after using a portion of the fluid for standard pathological procedure, but Ortea et al. used BALF as source for proteomic analysis and identified sensitive biomarkers by targeted proteomics DIA. They found forty-four proteins with a fold-change higher than 3.75 among ADC patients compared with controls where CO4A, GTSP1, and HPT are consistent with previous studies.

The major challenge for lung cancer therapy is chemoradioresistance, where protein markers might serve as the potential molecular predictors of drug resistance and overcome this shortage. Recent study of a SILAC-based quantitative proteomic approach has been utilized to evaluate the cellular protein abundance changes upon paclitaxel (PTX) treatment. Tumor suppressor programmed cell death 4 (PDCD4) in lung cancer tissues were positively correlated with the longer overall survival of lung cancer patients with PTX treatment, suggesting that PDCD4 may be used as a predictive marker of resistance to PTX in lung cancer patients [46]. Furthermore, SILAC-based quantitative proteomic strategy has been applied to reveal the functional role of invasiveness-associated KPNA2 protein complex in lung adenocarcinoma cell lines [47]. Integrating the omic data from DNA, RNA, and proteins data sets might represent new anticancer therapeutic targets for lung cancer. Li et al. integrated the genomic and proteomic data sets of lung cancer to construct omic map to represent non-small cell lung carcinoma [11]. In addition, a proteogenomic study of lung adenocarcinoma identified 565 proteins and 629 genes to be differentially expressed between SCC and ADC by TMT labeled quantitative proteomics, and suggested MCT1 and GLUT1 are the promising drug targets or histological marker [48].

## Discovery of therapeutic targets by quantitative proteomics

During the stages of drug development, proteomics can also take place in a high-throughput analysis for the identification and optimization of suitable lead compounds. Several tyrosine kinase inhibitors (TKIs) have been approved from the US Food and Drug Administration (FDA) for use in advanced lung cancer. Epidermal growth factor receptor (EGFR) and anaplastic lymphoma kinase (ALK) are the two common biological targets for lung cancer drug development [49, 50]. Application of these targeted therapies in selected patients has shown consistent benefits with regard to clinical outcomes [51]. Quantitative proteomics is able to predict for which patient might benefit from targeted therapy by understanding the molecular mechanism underneath (Table 3).

**Table 3:** Drug target and molecular mechanism in lung cancer

Target	Mechanism	Ref.
ALK	Alectinib (C_30_H_34_N_4_O_2_) is an inhibitor of ALK, which binds to and inhibits not only ALK kinase but also the L1196M mutant. Ceritinib (C_28_H_36_ClN_5_O_3_S) is a ATP-competitive, tyrosine kinase inhibitor of ALK, especially for *ALK*-rearranged NSCLC. Crizotinib (C_21_H_22_Cl_2_FN_5_O) is a kinase inhibitor for ALK, c-Met, and ROS1, especially for *ROS1*-rearranged NSCLC.	[59] [58] [60]
ATP synthase	Citreoviridin (C_23_H_30_O_6_) inhibits the mitochondrial ATP synthetase system. It has been used to target ectopic ATPase activity in lung cancer cells in order to modulate the metabolic activity associated with tumorigenesis.	[64]
BRAF	Vemurafenib (C_23_H_18_ClF_2_N_3_O_3_S) selectively binds to the ATP-binding site of BRAF (V600E) kinase, since most BARF gene mutations exist at residue 600 which has been found to be over-activates the MAPK signaling pathway.	[83]
EGFR	Afatinib (C_24_H_25_ClFN_5_O_3_) selectively inhibits ErbB1, ErbB2, ErbB4 and EGFR mutants (L858R and T790M). It may inhibit tumor progression and angiogenesis. Erlotinib (C_22_H_23_N_3_O_4_) is a protein kinase inhibitor which inhibits EGFR phosphorylation and blocks signal transduction. However, the FDA-approval is limited to EGFR mutations. Gefitnib (C_22_H_24_ClFN_4_O_3_) inhibits the catalytic activity of tyrosine kinase that competes with the binding affinity of ATP to the tyrosine kinase domain of EGFR. It inhibits signal transduction by inhibiting the receptor for phosphorylation.	[55] [54] [53]

EGFR has become an important biological target for lung cancer. Inhibitors that target EGFR and block the signaling pathway have been developed and are clinically active [52]. Three EGFR inhibitors including afatinib, erlotinib, and gefitinib are used in NSCLC with EGFR mutated patients. Gefitnib inhibits the catalytic activity of tyrosine kinase that competes with the binding affinity of ATP to the tyrosine kinase domain of EGFR [53]. It inhibits signal transduction by inhibiting the receptor for phosphorylation. Erlotinib is a protein kinase inhibitor which inhibits EGFR phosphorylation and blocks signal transduction [54]. However, the FDA-approval is limited to EGFR mutations. For lung cancer patients who has EGFR mutation, an initial treatment with EGFR TKI is preferred. Afatinib selectively inhibits ErbB1, ErbB2, ErbB4 and especially EGFR mutants (L858R and T790M) which inhibits tumor progression as well as angiogenesis [55]. Comparative proteome profiling across 23 NSCLC cell lines revealed the significant expression differences in cell lines harboring oncogenic KRAS and EGFR mutations [56]. This study provided valuable information for the identification of candidate therapeutic targets, which mediate oncogenic processes driven by K-Ras or EGFR mutant protein expression. A multicohort cross-institutional study performed by Taguchi et al., they classified NSCLC patients for clinical outcome after treatment with EGFR TKI by mass spectrometry. Serum collected from 139 NSCLC patients were analyzed by mass spectrometry which might provide valuable resources for clinical benefit from a molecularly anticancer drug [57].

Chromosomal rearrangements of ALK have been found to be associated with lung cancer and its inhibitors ceritinib are superior for patients with chemotherapy [49]. Ceritinib is a ATP-competitive, tyrosine kinase inhibitor of ALK, especially for ALK-rearranged NSCLC [58]. Alectinib is also an inhibitor of ALK, which binds to and inhibits not only ALK kinase but also the L1196M mutant [59]. Furthermore, crizotinib is a kinase inhibitor for multiple lung cancer oncogene including ALK, c-Met, and ROS1, especially for ROS1-rearranged NSCLC [60]. Current diagnostic test for ALK arrangement is based on low throughput fluorescence in situ hybridization (FISH), Hembrough et al. developed an ALK protein assay that could save time and the expense of multiple FISH testing to detect different biomarkers [61]. They used SRM approach to quantify absolute amounts of ALK in 188 formalin-fixed paraffin-embedded NSCLC tissues and the results were correlated with patients response to crizotinib.

Our recent study demonstrated ectopic ATP synthase that presents on the plasma membrane of lung cancer cells is a potential biological target for drug development [62, 63]. Citreoviridin serves as ATP synthase inhibitor which selectively suppresses the proliferation and growth of lung cancer without affecting normal cells [63]. Comprehensive proteomics were also performed using lung tumor xenografts treat with citreovirdin that reveals its antitumorigenic effects in lung cancer, which may lead to a better understanding of the links between metabolism and tumorigenesis in lung cancer drug development [62, 64].

## Quantitative proteomics enables to link proteins into functional networks

Modern high-throughput technologies generate a huge amount of data, however, proper data mining tools is the key for discovery of cancer-related proteins and networks. Fully understanding of the biological significance of differential protein networks from normal to disease cells depends on the information generated from proteomic datasets. Exploration of proteomic datasets using bioinformatic analysis enables us to elucidate new molecular interactions, protein functional annotation, protein motif and complex interaction and disease pathway.

With the advent of high-throughput omics data, bioinformatics has become a viable tool to improve our knowledge of health and disease individuals and it also provides a systems-level approach to interpret organisms and functional activities of their components by studying underneath interactions. Bioinformatics can be defined as a combination of mathematical and computational strategies for interpreting biological processes from the existing raw data. Data curation, tool development, and practical applications are the three major aims for bioinformatics [65]. To date, many biological databases are standardized and annotated for researchers to access existing information and also to submit new entries. Biological databases consist the information for protein sequencing (Uniprot, Swiss-Prot, Pfam), proteomics (PRIDE, ProteomeScout, OWL), protein structure (PDB, SCOP), and protein model (Swiss-model, SIMAP).

Mathematical and statistical approaches have become essential components for bioinformatics tool development. For example, developing a tool for protein structure requires serious consideration of the primary protein sequence, differential geometry and topology of the protein folding regardless of its biological functions. It is painstaking task for experimental biologists to interpret their dataset without bioinformatics tools, therefore, bioinformatics tool development takes an important part of proteomic and biological researches.

## Protein-protein interaction networks

Protein-protein interaction (PPI) networks provide an overall picture for the understanding of biological processes in cancer research. Proteins are not functioning solely, they have interactions with other proteins or molecules that mediate signaling pathways and biological processes. Hub proteins are highly connected to other proteins in a network, whereas some others have few interactions [66]. The dysfunction of protein-protein interactions is one of the causes for many diseases, including cancer [67]. Therefore, cancer can be enlightened through protein interaction networks, which in turn can appraise methods for cancer prevention, early diagnosis, and drug discovery. Many web-based resources such as STRING and Reactome are available for functional protein association network and signal transduction pathway analyses.

A dynamic PPI network of lung cancer associated with smoking was constructed by bioinformatic analysis using Human Protein Reference Database and Gene Expression Omnibus Database. Yu et al. used the support vector machine (SVM) model and found 520 dynamic proteins and 2754 static proteins and further predicted 7 dynamic PPI subnetworks for lung cancer patients with smoking history [68].

## Mathematical modeling

**Fig. 2:**
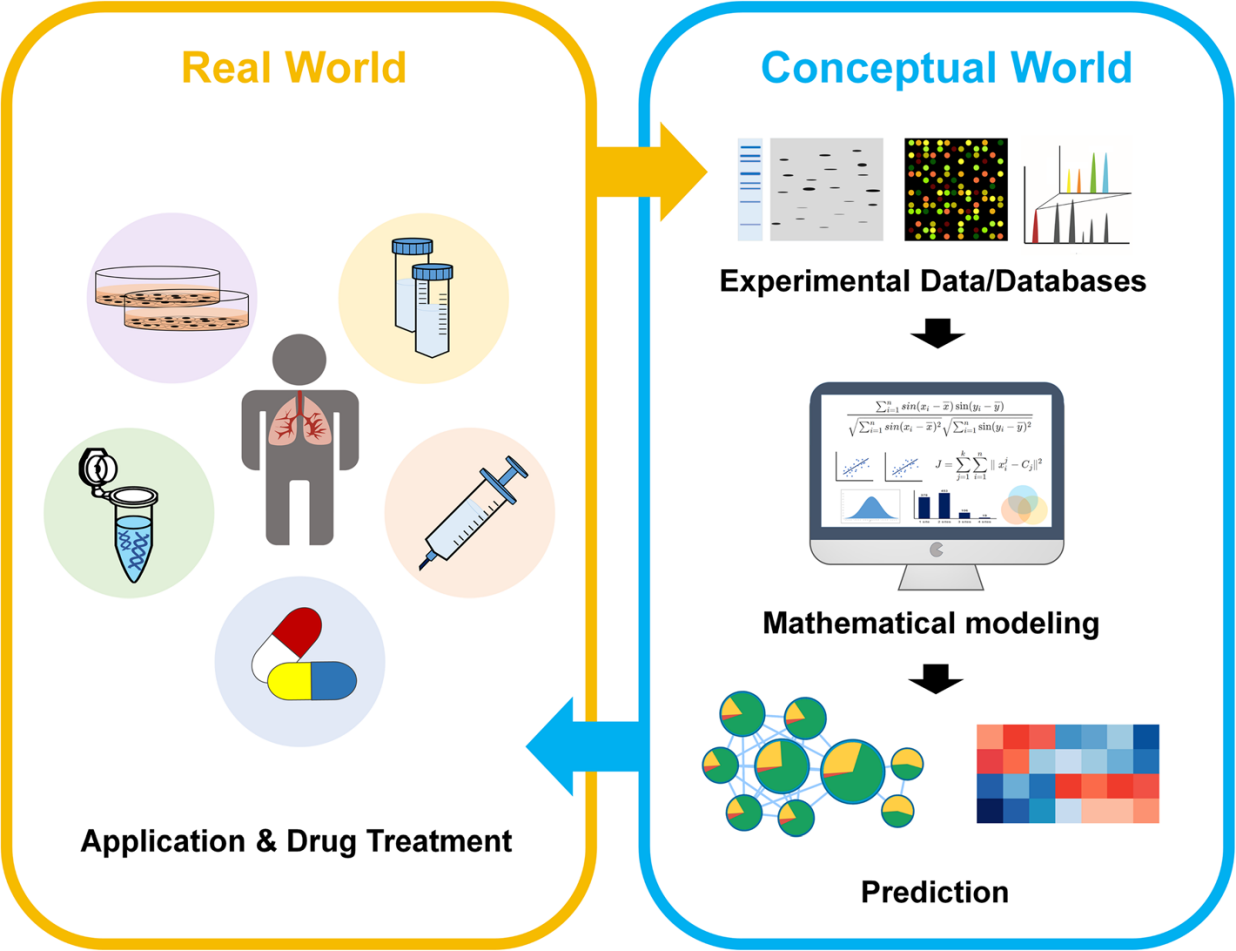
The depiction of the mathematical modeling in the conceptual world to the real world. Mathematical modeling empowers the researchers to examine the relationship between the biological processes in the real world and the predictions in the conceptual world. With the advent of high-throughput omics data, bioinformatics and mathematical modeling have become viable tools to improve our knowledge of molecular mechanism of cancer related phenomenon. It is a computational simulation that applied mathematical approaches of quantitative calculation for hundreds of components and their interactions and thus have the potential of truly explanation for complex diseases such as lung cancer. Researchers are able to systematically investigate systems perturbations, develop hypotheses to design new experiments, and ultimately predict the reliable candidates as novel therapeutic targets

Mathematical modeling is a time and cost-effective method that provides insight into underlying molecular reactions and biological processes as alternatives to conventional laboratory experiments [69]. Mathematical modeling empowers the researchers to examine the relationship between the biological processes in the real world and the predictions in the conceptual world (Fig. 2). It is a computational simulation tool that utilizes mathematical approaches of quantitative calculation for hundreds of components and their interactions and thus have the potential of truly explanation for complex diseases such as cancer [70]. Researchers are able to systematically investigate systems perturbations, develop hypotheses to design new experiments, and ultimately predict the reliable candidates as novel therapeutic targets [71].

Chmielecki et al. developed isogenic TKI-sensitive and TKI-resistant pairs of cell line that mimic the behavior of NSCLC with evolutionary cancer modeling [72]. They combined in vitro experiments, multiple clinical data sets, and mathematical modeling to describe NSCLC behavior. Their mathematical modeling proposed that alternative therapeutic strategies could prolong the benefit of TKI against EGFR-mutant lung cancer by delaying the development of resistance. Our recent study of a dynamic network in response to an ATP synthase inhibitor citreovirdin by mathematical modeling and bioinformatics analysis revealed that citreoviridin suppresses lung cancer cell growth via mitogen-activated protein kinase signaling by dephosphorylation of heat shock protein 90 β on Serine 255 [62]. Construction of protein networks provides an opportunity to interpret disease pathway and improve our understanding in cancer therapeutic strategies.

## Conclusions

Since lung cancer is a heterogeneous disease, a comprehensive and in-depth discovery of lung cancer proteomic profiling is needed for precise target treatment. The microenvironment interface of the tumor cells and host cells directly impacts the tumor-host communication system by affecting signaling and growth factors, therefore cancer processing [73]. To understand the biological significance of differential protein networks from normal to disease cells depends on the proteomic datasets, where new molecular interactions, protein functional annotation, protein motif and complex interaction and disease pathway are able to analyze by bioinformatics analysis. Furthermore, the functional diversity of proteins is generated by post-transcriptional modifications (PTM) such as phosphorylation, acetylation, and ubiquitination. To characterize the post-transcriptional modifications might expand our knowledge of dynamic system in cancer progression. The majority of PTM research for proteomics are shotgun proteomics; however, the complexity of proteomics datasets requires standards to ensure reproducibility and unambiguous interpretation. An alternative method using targeted proteomics by SRM and MRM with sensitive detection enable us to identify specific PTM and give the absolute copy number of proteins in a single cell. Recent study of phosphorylation dynamics in non-small cell lung cancer by targeted proteomics including SRM, MRM, and DIA enabled the quantification of 42 PI3K-mTOR and MAPK phosphosites and provides valuable conclusion on each assessment [74]. Quantitative proteomics provides the information for synthetic biologists to engineer or rewire the key pathways, furthermore to offers the best therapeutic strategy for lung cancer.

## Abbreviations

1D: One-dimensional
2D: Two-dimensional
ADC: Adenocarcinoma
ALK: Anaplastic lymphoma kinase
ATP: Adenosine triphosphate
BALF: Bronchoalveolar lavage fluid
BRAF: Serine/threonine-protein kinase B-raf
DIA: Data-independent acquisition
DIGE: Difference gel electrophoresis
EGFR: Epidermal growth factor receptor
FDA: The US Food and Drug Administration
iTRAQ: Isobaric tags for relative and absolute quantification
m/z: Mass-to-charge ratio
MET: Hepatocyte growth factor receptor
Mr: Molecular weight
MRM: Multiple reaction monitoring
MS: Mass spectrometry
NSCLC: Non-small cell lung cancer
PE: Pleural effusion
pI: Isoelectric point
PTX: Paclitaxel
ROS1: Proto-oncogene tyrosine-protein kinase ROS
SCC: Squamous cell carcinoma
SCLC: Small cell lung cancer
SILAC: Stable isotopic labeling with amino acids in cell culture
SILAM: Stable isotope labeling in mammals
SRM: Selected reaction monitoring
TKI: Tyrosine kinase inhibitors
